# Quantitative trait variation in ASD probands and toddler sibling outcomes at 24 months

**DOI:** 10.1186/s11689-020-9308-7

**Published:** 2020-02-05

**Authors:** Jessica B. Girault, Meghan R. Swanson, Shoba S. Meera, Rebecca L. Grzadzinski, Mark D. Shen, Catherine A. Burrows, Jason J. Wolff, Juhi Pandey, Tanya St John, Annette Estes, Lonnie Zwaigenbaum, Kelly N. Botteron, Heather C. Hazlett, Stephen R. Dager, Robert T. Schultz, John N. Constantino, Joseph Piven, for the IBIS Network, C. Chappell, D. Shaw, J. Elison, M. Styner, G. Gerig, R. McKinstry, J. Pruett, A. C. Evans, D. L. Collins, V. Fonov, L. MacIntyre, S. Das, H. Gu, K. Truong, H. Volk, D. Fallin

**Affiliations:** 1 Carolina Institute for Developmental Disabilities, University of North Carolina at Chapel Hill, Campus Box 3376, Chapel Hill, NC 27599 USA; 2 Department of Psychology, School of Behavioral and Brain Sciences, University of Texas at Dallas, Richardson, TX USA; 3 National Institute of Mental Health and Neurosciences, Bangalore, India; 4 Department of Psychiatry, University of North Carolina at Chapel Hill, Chapel Hill, NC USA; 5 Department of Pediatrics, University of Minnesota, Minneapolis, MN USA; 6 Department of Educational Psychology, University of Minnesota, Minneapolis, MN USA; 7 Center for Autism Research, Children’s Hospital of Philadelphia, University of Pennsylvania Perelman School of Medicine, Philadelphia, PA USA; 8 Department of Speech and Hearing Science, University of Washington, Seattle, WA USA; 9 Department of Pediatrics, University of Alberta, Edmonton, AB Canada; 10 Mallinckrodt Institute of Radiology, Washington University School of Medicine, St. Louis, MO USA; 11 Department of Radiology, University of Washington Medical Center, Seattle, WA USA; 12 Division of Child Psychiatry, Department of Psychiatry, Washington University School of Medicine, St. Louis, MO USA

**Keywords:** Autism, Infant sibling, Family study, Language, Communication, Development

## Abstract

**Background:**

Younger siblings of children with autism spectrum disorder (ASD) are at increased likelihood of receiving an ASD diagnosis and exhibiting other developmental concerns. It is unknown how quantitative variation in ASD traits and broader developmental domains in older siblings with ASD (probands) may inform outcomes in their younger siblings.

**Methods:**

Participants included 385 pairs of toddler siblings and probands from the Infant Brain Imaging Study. ASD probands (mean age 5.5 years, range 1.7 to 15.5 years) were phenotyped using the Autism Diagnostic Interview-Revised (ADI-R), the Social Communication Questionnaire (SCQ), and the Vineland Adaptive Behavior Scales, Second Edition (VABS-II). Siblings were assessed using the ADI-R, VABS-II, Mullen Scales of Early Learning (MSEL), and Autism Diagnostic Observation Schedule (ADOS) and received a clinical best estimate diagnosis at 24 months using DSM-IV-TR criteria (*n* = 89 concordant for ASD; *n* = 296 discordant). We addressed two aims: (1) to determine whether proband characteristics are predictive of recurrence in siblings and (2) to assess associations between proband traits and sibling dimensional outcomes at 24 months.

**Results:**

Regarding recurrence risk, proband SCQ scores were found to significantly predict sibling 24-month diagnostic outcome (OR for a 1-point increase in SCQ = 1.06; 95% CI = 1.01, 1.12). Regarding quantitative trait associations, we found no significant correlations in ASD traits among proband-sibling pairs. However, quantitative variation in proband adaptive behavior, communication, and expressive and receptive language was significantly associated with sibling outcomes in the same domains; proband scores explained 9–18% of the variation in cognition and behavior in siblings with ASD. Receptive language was particularly strongly associated in concordant pairs (ICC = 0.50, *p* < 0.001).

**Conclusions:**

Proband ASD symptomology, indexed by the SCQ, is a predictor of familial ASD recurrence risk. While quantitative variation in social communication and restricted and repetitive behavior were not associated among sibling pairs, standardized ratings of proband language and communication explained significant variation in the same domains in the sibling at 24 months, especially among toddlers with an ASD diagnosis. These data suggest that proband characteristics can alert clinicians to areas of developmental concern for young children with familial risk for ASD.

## Introduction

Autism spectrum disorder (ASD) is a highly heritable [1] neurodevelopmental disorder diagnosed in 1–2% of children [2]. While significant advances in genetics have identified de novo mutations in a portion of the ASD population, the vast majority of ASD cases are attributable to common [3, 4], additive [3, 5] polygenic variation. The heritable nature of ASD is reflected in the recurrence risk in families, where prospective, longitudinal studies of infant siblings of older children with ASD (probands) have revealed that approximately 20% of high-risk younger siblings receive a diagnosis themselves [6]. An additional 28% of high-risk siblings who do not meet the diagnostic criteria for ASD exhibit atypical behavioral profiles in toddlerhood [7], suggesting an important role for ASD genetic liability in child development more broadly.

Studies in biological siblings have revealed important insights into the heritability of ASD traits and broader developmental domains in families affected by ASD. Among sibling and twin pairs with ASD or subthreshold ASD symptomology, similarities are present in the domains of socialization [8–10], communication [8–10], and adaptive behavior [9, 10]. Other studies have reported phenotypic congruence in cognitive performance, including language skills and verbal and nonverbal communication abilities, among twin and sibling pairs concordant for ASD [8, 9, 11–13]. While fewer large-scale studies have investigated the familial aggregation of ASD symptom domains, there is evidence that nonverbal communication and social impairments are correlated among affected sibling pairs [8, 14], with nonverbal communication being most heritable [15]. Studies of symptom profiles in sibling pairs have not found restrictive and repetitive behaviors to be similar among siblings [8, 9, 11]. This work has provided largely convergent evidence that the *level* of cognitive and behavioral functioning in siblings with ASD is familial in nature.

In the context of ASD recurrence in families, it becomes critical to understand how characteristics of the proband—as indices of a potentially shared genetic liability for ASD—may inform diagnostic and developmental outcomes in their younger sibling(s) during a period suitable to early intervention. The prospective nature of the infant sibling study design is poised to address these questions, though only two studies of this kind have been reported to date. Schwichtenberg and colleagues [16] investigated whether social-communicative features of first-degree family members informed infant sibling categorical outcome (ASD, atypical development, typical development) at 36 months, and found no significant association between the parent or proband-autistic traits and infant sibling categorical outcome group. Similarly, Ozonoff and colleagues [6] reported that ASD social communication in probands was not predictive of ASD diagnostic outcome group (ASD vs. no ASD) in younger siblings. These studies found that proband ASD-related social communication abilities were not predictive of categorical or diagnostic outcomes in younger siblings, though it remains unclear whether other proband traits (repetitive behaviors, adaptive behaviors) may inform recurrence risk. Further, there have been no investigations relating proband traits to continuous, *quantitative* variations in ASD symptomology or other behavioral traits in toddler siblings. Thus, it is unknown whether proband traits hold predictive power for specific areas of developmental concern, beyond diagnostic outcome, in younger siblings from high-risk families.

In the present study, we leveraged the prospective longitudinal design of the Infant Brain Imaging Study (IBIS) to address two primary research goals: (1) to determine whether proband characteristics are predictive of ASD recurrence in their younger sibling and (2) to assess the extent to which proband traits explain variation in toddler sibling dimensional outcomes at 24 months. We focused on the defining features of ASD and domains shown to be associated among older sibling pairs, including adaptive behavior, socialization, communication, and repetitive behaviors. We also investigated domains shown to be aberrant during the first 2 years of life in high-risk siblings, including motor and language abilities [7, 17–21], which may serve as targets for early intervention.

## Methods

### Participant sample

IBIS is an ongoing, longitudinal study of infants at familial risk for ASD by virtue of having an older sibling with a diagnosis of ASD, verified by medical records and the Autism Diagnostic Interview-Revised (ADI-R); any additional older siblings with ASD were not phenotyped. All participants were screened and excluded based on the following criteria: (1) known genetic conditions or syndromes in the proband or infant; (2) medical/neurological conditions affecting growth, development, or cognition (e.g., vision or hearing loss); (3) birth weight < 2000 g and/or gestational age < 36 weeks or significant perinatal adversity and/or exposure to in utero neurotoxins; (4) contraindication for MRI; (5) predominant home language other than English; (6) adopted children or half siblings; (7) first-degree relative with psychosis, schizophrenia, or bipolar disorder screened using the Family Interview for Genetic Studies [22]; and (8) multiple gestation pregnancy. Parents provided written informed consent prior to participating in this study. Procedures for this study were approved by the Institutional Review Boards at each clinical data collection site: University of North Carolina at Chapel Hill, University of Washington in Seattle, Children’s Hospital of Philadelphia, and Washington University in St. Louis. Data coordination was managed by the Montreal Neurological Institute at McGill University. A comparison sample of infants with typically developing older siblings was included in the larger IBIS study; however, parental interviews on typically developing older siblings’ adaptive behavior were not collected, and thus, these sibling pairs were not included here.

The present study included 385 pairs of familial high-risk toddlers and their older siblings with ASD (proband). Behavioral data were available for both the toddler and the proband on at least 1 parent interview or examiner-based assessment (sample sizes per assessment are shown in Table 1), and a diagnostic outcome was available for the toddler sibling at 24 months. A total of 89 sibling pairs were concordant for ASD based on toddler sibling diagnosis of ASD at 24 months. The remaining 296 pairs were discordant for ASD, as the younger siblings did not receive an ASD diagnosis. The sample characteristics are reported in Table 1.

**Table 1:** Participant characteristics and sample sizes

	Probands (*n* = 385)		Siblings					
			ASD (*n* = 89)		No-ASD (*n* = 296)		Chi square^a^	
	*n*	%	*n*	%	*n*	%	*χ*^2^	*p*
Sex							14.69	0.0001
Female	53	13.8	20	22.5	136	46.0		
Male	332	86.2	69	77.5	160	54.0		
Child race							10.97	0.6887
Asian	3	0.8	1	1.1	3	1.0		
Black	12	3.1	3	3.4	10	3.4		
More than one race	36	9.4	13	14.6	26	8.8		
White	300	77.9	69	69.7	233	78.7		
Not answered	34	8.8	10	11.2	24	8.1		
Maternal education							4.03	0.1336
No college	125	32.5	36	40.5	91	30.7		
College degree	153	39.7	28	31.5	125	42.2		
Graduate degree	94	24.4	23	25.8	71	24.0		
Missing	11	2.86	2	2.2	9	3.04		
Number of assessments^b^								
ADI-R	372	96.6	86	96.6	281	94.9		
ADOS	–	–	75	84.3	261	88.2		
MSEL	–	–	87	97.8	295	99.7		
SCQ	346	89.9	–	–	–	–		
VABS-II	331	86.0	85	95.5	291	98.3		

^a^Comparison between siblings with and without a diagnosis of ASD

^b^Number of participants with at least one of the scales of interest from each assessment

### Diagnostic classification

Clinical best estimate diagnoses were made at 24-month visits by experienced, licensed clinicians using the DSM-IV-TR criteria for autistic disorder or pervasive developmental disorder, not otherwise specified, and collectively referred to as ASD. The DSM-IV was used for diagnostic classification as the DSM-5 was released in the later phases of the IBIS study. A complete description of the assessment and diagnostic procedures is reported by Estes et al. [17].

### Clinical and behavioral measures

A list of corresponding proband and toddler sibling measures are reported in Table 2. Proband measures were collected using parent interviews including the ADI-R, Social Communication Questionnaire (SCQ), and Vineland Adaptive Behavior Scales, Second Edition (VABS-II). Proband behavioral data were largely collected at the younger sibling’s first visit as part of the larger longitudinal study at 6 months of age, but some variation in timing for data collection (i.e., parent interviews on the proband taken at a subsequent study visit) resulted in slightly different age ranges for each proband measure (ADI-R: mean age 5.5 years, range 1.9 to 15.5 years; SCQ: mean age 5.5 years, range 1.7 to 15.5 years; VABS-II: 5.6 years, range 1.8 to 15.5 years). The proband’s chronological age at the collection of each respective parent interview was entered as a covariate in statistical analyses. Toddler sibling scores included a combination of parent interviews and examiner-based assessments including the ADI-R, Autism Diagnostic Observation Schedule (ADOS), VABS-II, and Mullen Scales of Early Learning (MSEL). All sibling data were collected at the 24-month visit (mean age = 24.7 months, SD = 0.59 months), and chronological age is included in all statistical models. Measures of interest from each assessment are described below.

**Table 2:** Behavioral and clinical measures of interest

Domain(s)	Probands	Siblings	
	Parent interview	Parent interview	Examiner-based
Autism traits	ADI-R, SCQ	ADI-R	ADOS
General ability/adaptive behavior	VABS-II: ABC, SOC	VABS-II: ABC, SOC	MSEL: ELC
Communication/language	VABS-II: COM, EL, RL	VABS-II: COM, EL, RL	MSEL: EL, RL
Motor	VABS-II: MS, GM, FM	VABS-II: MS, GM, FM	MSEL: GM, FM

*ADI-R* Assessments include the Autism Diagnostic Interview-Revised, *ADOS* Autism Diagnostic Observation Schedule, *SCQ* Social Communication Questionnaire, *VABS-II* Vineland Adaptive Behavior Scales Second Edition, and the *MSEL* Mullen Scales of Early Learning

Measures of interest from the MSEL and VABS-II include *ABC* adaptive behavior composite, *ELC* Early Learning Composite, *SOC* socialization, *COM* communication, *EL*, *RL* expressive and receptive language, *MS* motor skills, *GM*, *FM* gross and fine motor

Higher scores on the ADI-R, SCQ, and ADOS indicate greater endorsement of ASD symptoms: higher scores on the VABS-II and MSEL indicate better adaptive and cognitive skills

The ADI-R is a diagnostic interview assessing the qualitative abnormalities in reciprocal social interaction and communication, restricted and repetitive behaviors, and the onset of atypical development at or before 36 months [23]. Measures of interest included the verbal and nonverbal communication, restricted and repetitive behavior (RRB), and social standard scores. Higher scores on the ADI-R reflect a greater endorsement of ASD symptomology. The ADI-R was administered by a research-reliable examiner, with only nonverbal items administered to nonverbal children. Of the 372 probands and 367 siblings with ADI-R data, 28% of probands (*n* = 105) and 57% of siblings (*n* = 211) were scored according to the nonverbal algorithm, and 72% of probands (*n* = 267) and 43% of siblings (*n* = 156) were scored using the verbal algorithm. Of the 355 sibling pairs with available ADI-R data, 118 pairs (33%) were scored using the verbal algorithm, 66 pairs (19%) were scored using the nonverbal algorithm (as shown in Table 3), and the remaining pairs (*n* = 171, 48%) were scored using opposite algorithms and are thus not compared with the analyses of the ADI-R data described below. Age-appropriate scoring algorithms were used; probands under the age of 4 years and siblings at the 24-month visit were scored using the algorithm validated for ages 2 years to 3 years and 11 months. Probands ages 4 years and older were scored using the standard algorithm. Distributions of proband and sibling ADI-R scores can be seen in Additional file 1: Figure S1 in the online supplemental material.

**Table 3:** Intraclass correlation coefficients

	All sibling pairs			Concordant pairs			Discordant pairs		
	*n*	ICC	*p*	*n*	ICC	*p*	*n*	ICC	*p*
ADI-R									
Social	355	0.03	0.096	81	− 0.20	0.595	274	0.02	0.112
RRB	355	0.03	0.056	81	0.02	0.386	274	0.04	0.027
Nonverbal communication	66	0.12	0.041	29	0.28	0.038	37	0.07	0.043
Verbal communication	118	0.04	0.087	18	0.08	0.209	100	0.01	0.389
VABS-II									
ABC	306	0.13	< 0.001^+^	65	0.27	0.005	241	0.09	0.008
Socialization	314	0.08	0.005	69	0.25	0.002^+^	245	0.03	0.207
Communication	313	0.22	< 0.001^+^	69	0.41	0.002^+^	244	0.14	0.006
Expressive	321	0.15	< 0.001^+^	71	0.35	0.001^+^	250	0.07	0.052
Receptive	322	0.23	< 0.001^+^	71	0.50	< 0.001^+^	251	0.12	0.008
Motor	281	0.11	0.027	62	0.05	0.344	219	0.11	0.053
Fine	282	0.09	0.094	61	0.00	0.506	221	0.09	0.125
Gross	281	0.21	< 0.001^+^	62	0.25	0.053	219	0.18	0.001^+^

^+^significant at *p* ≤ 0.004 after Bonferroni correction (12 comparisons per group)

The SCQ, derived from the original ADI, is a 40-item parent-report screening instrument for ASD that focuses on items relating to ASD symptomology likely to be observed by a primary caregiver [24]. This study utilized the SCQ Lifetime version referencing complete developmental history (past and present) of the proband, with a subset of items focused on the period of time between the proband’s fourth and fifth birthdays; if the proband was not yet 4 years, parents were asked to report on the past 12 months. The SCQ was not administered to siblings at the 24-month visit given the generally limited validation of the SCQ in populations under 30 months of age [25]. SCQ total scores range from 0 to 33 for nonverbal children and from 0 to 39 for verbal children. In our sample of 348 probands with SCQ data, 24% (*n* = 83) were scored using the nonverbal algorithm and 76% (*n* = 265) were scored using the verbal algorithm. Higher scores on the SCQ reflect a greater endorsement of ASD symptomology. Proband SCQ distributions are shown in Additional file 1: Figure S1.

The VABS-II provides measures of adaptive behavior in everyday settings and includes assessment of communication, daily living, and social and motor skills [26]. For this study, we utilized the Adaptive Behavior Composite (ABC), the socialization (SOC), communication (COM), and motor skills (MS) standard scores. The scale scores of expressive and receptive language (EL, RL) and fine and gross motor (FM, GM) were also examined. The ABC, SOC, COM, and MS standard scores range from 20 to 160 (mean = 100, SD = 15), and scale scores (EL, RL, FM, GM) range from 1 to 24 (mean = 15, SD = 3), where higher scores indicate better adaptive skills.

Toddler sibling measures of interest at 24 months included the same parent-reported measures from the VABS-II, as well as examiner-based assessments of similar constructs on the Mullen Scales of Early Learning [27]. Specifically, the Early Learning Composite (ELC) standard score and scale *T*-scores corresponding to domains measured in probands using the VABS-II: GM, FM, EL, and RL. The ELC ranges from 49 to 155 (mean = 100, SD = 15), and *T*-scores range from 20 to 80 (mean = 50, SD = 10) [27]; higher scores indicate better cognitive skills. Autism traits were assessed using the ADI-R and the ADOS [28]. The ADOS is a semi-structured play assessment of the characteristic features of ASD, capturing communication, social interaction, play skills, and RRBs. A research-reliable evaluator administered the ADOS module 1 or 2 (depending on language level) to siblings at the 24-month visit. ADOS scores of interest included the overall calibrated severity score [29] and the calibrated severity score for social affect (SA) [30]. ADOS calibrated severity scores range from 1 to 10, where higher scores indicate greater endorsement of ASD symptoms. As with probands, the verbal and nonverbal communication, RRB, and social scores from the ADI-R were used.

### Statistical analyses

First, we sought to determine how proband ASD traits related to toddler sibling diagnostic outcomes. ANCOVA was used to test whether probands of concordant and discordant pairs differed in terms of their ASD trait level. Sibling diagnostic group was entered as a categorical independent variable, controlling for proband sex and age; primary dependent variables were proband SCQ total score and ADI-R social, RRB, and communication scores. Secondary analyses were performed to test for group differences in proband ABC, SOC, COM, and MS composite scores from the VABS-II. Proband scores shown to significantly differ between concordant and discordant pairs were entered as independent variables in a logistic regression analysis predicting sibling diagnostic outcome, controlling for age at assessment for the proband and sibling, sex of the proband and sibling, and clinical study site.

In order to determine the extent to which variation in ASD traits, adaptive behavior, socialization, communication and language, and motor skills are associated among sibling pairs, intraclass correlation coefficients (ICCs; two-way mixed, absolute agreement, average measure) accounting for the clustered nature of the data (i.e., siblings grouped as pairs) were calculated for identical measures (ADI-R, VABS-II). In this case, ICCs offer the advantage over Pearson correlations by taking into account the *agreement* of scores among sibling pairs, not simply linear associations. We used the results of the ICC analysis to identify variables of interest for further investigation, where any measures or domains found to be significantly correlated were retained for regression analyses.

Next, linear regression analyses were performed, where primary independent variables included proband scores from the VABS-II and dependent variables included both parent report of sibling behavior on the VABS-II and examiner-based measures of similar constructs on the MSEL at 24-months. This allowed us to ensure that our findings were not an artifact of comparing parent reports across sibling pairs. All models included proband and sibling age and sex, study site, and sibling diagnostic group as covariates. A proband score by sibling diagnostic group interaction term was included to test the hypothesis that the predictive ability of proband traits for sibling behavior is stronger in concordant pairs. All linear regression coefficients are standardized, and bivariate Pearson correlations were calculated for significant linear regression models to aid in the interpretation of the effect sizes across measures (ICCs cannot be calculated across different measures). We confirmed that model assumptions were met for normality and heteroscedasticity using quantile-quantile plots and by plotting the associations between fitted values and residuals, respectively, for all linear models.

Several analyses were conducted to evaluate the robustness of the results. To ensure our results are not impacted by opposite-sex sibling pairs, primary models were re-analyzed with only male-male sibling pairs (*n* = 206). Due to the small number of female-female sibling pairs (*n* = 30 total, *n* = 7 concordant for ASD), we did not perform analyses on female-only pairs. In an effort to identify and filter out probands with potential intellectual disability that may be due to de novo mutations and not inherited genetic variation [31], we conducted analyses excluding pairs where the proband scored < 60 on the VABS-II ABC (*n* = 286), translating to performing below the first percentile. While adaptive behavior and intellectual ability are not interchangeable, these domains are more highly correlated in individuals with ASD and comorbid intellectual disability [32], thus, this approach is conservative in identifying probands with intellectual disability. We also tested the effects of maternal education level (less than a college degree, college degree, graduate degree) on our models. Finally, due to several toddlers scoring at the floor on the MSEL EL and RL *T*-scores, we re-analyzed the MSEL data using age-equivalent scores to avoid a skew in the distribution.

Linear and logistic regressions, ANCOVAs, *t* tests, Cohen’s *d* effect sizes, and Pearson correlations were performed using R version 3.5.1; ICCs were computed using IBM SPSS Statistics version 26. Bonferroni correction was applied to each analysis to adjust for the number of comparisons of interest.

## Results

### Proband traits as predictors of sibling diagnostic outcomes

ANCOVA models revealed that probands of concordant pairs scored higher on the SCQ than probands of discordant pairs (*F*_1342_ = 4.89, *p* = 0.028, Cohen’s *d* = 0.27). Proband scores on the ADI-R (social: *F*_1368_ = 1.28, *p* = 0.259; RRB: *F*_1368_ = 0.879, *p* = 0.349; nonverbal communication: *F*_1101_ = 1.34, *p* = 0.250; verbal communication: *F*_1263_ = 2.02, *p* = 0.157) and composite scores from the VABS-II (ABC: *F*_1319_ = 1.77, *p* = 0.185; SOC: *F*_1323_ = 2.63, *p* = 0.104; COM: *F*_1322_ = 2.59, *p* = 0.109; MS: *F*_1290_ = 0.793, *p* = 0.374) were not significantly different between probands of concordant and discordant pairs.

Proband SCQ total score was then entered into a logistic regression predicting sibling diagnostic outcome, along with proband and sibling age and sex, and study site. As expected, based on previous reports [6], sex of the toddler sibling significantly predicted diagnostic outcome (*β* = 1.21, SE = 0.38, *p* = 0.0001; OR for males = 3.34; 95% CI = 1.19, 6.36). Above and beyond the sex of the sibling, we found that proband ASD symptomology indexed by the SCQ total score significantly predicted sibling diagnostic outcome at 24 months (*β* = − 0.06, SE = 0.026, *p* = 0.014; OR for a 1-point increase in SCQ = 1.06; 95% CI = 1.01, 1.12). For each additional point a proband scored on the SCQ—reflective of the endorsement of additional ASD symptoms—the odds of the toddler sibling receiving a diagnosis of ASD increased by 6%. Other proband characteristics including chronological age (OR = 0.99; CI = 0.98, 1.0) and sex (OR for males = 0.71; CI = 0.34, 1.54) did not significantly predict sibling diagnostic outcomes. In supplemental analyses, SCQ results were probed further by splitting the proband sample into quartiles and controlling for the verbal and nonverbal status of the proband. Findings from all analyses suggest the SCQ is a significant predictor of toddler sibling diagnostic outcomes at 24 months, see Additional file 1: Tables S1–S2 in the online supplemental material for full model results.

### Proband-sibling associations: ASD traits

For ASD traits measured by the ADI-R, associations between proband-sibling pairs were generally weak and none survived correction for multiple comparisons (Table 3). Though, when comparing the ICCs for pairs concordant for ASD, the correlation for abnormalities in communication is notably higher (ICC = 0.28) among nonverbal pairs than verbal pairs (ICC = 0.08). We also tested for cross-instrument correlations between proband SCQ scores and sibling ADOS calibrated severity scores as an additional evaluation of ASD trait similarities among sibling pairs; we found no significant associations (ADOS social: *r* = − 0.19, *p* = 0.123; ADOS calibrated severity: *r* = − 0.08, *p* = 0.455).

### Proband-sibling associations: cognition and behavior

**Fig. 1:**
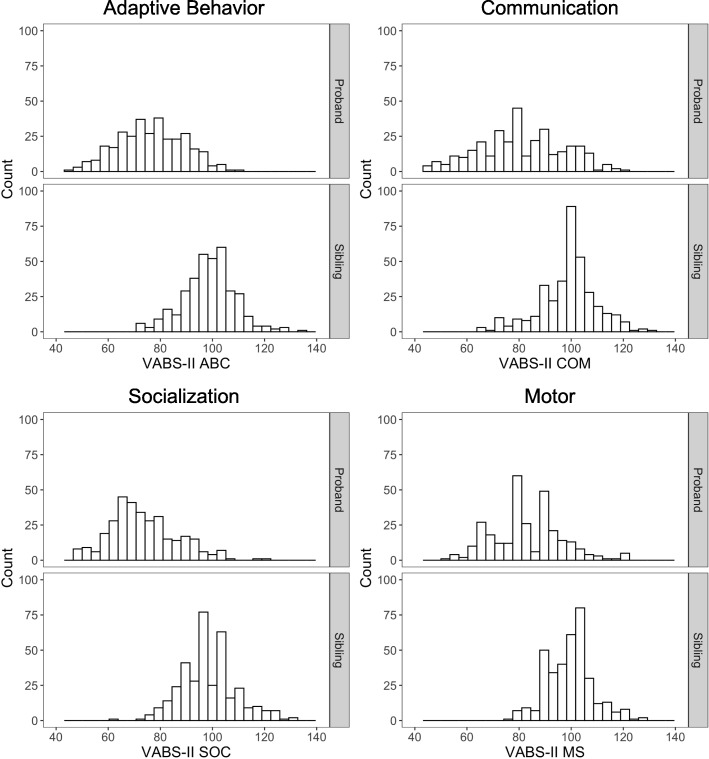
Proband and sibling VABS-II composite score distributions. Histograms display a wide distribution of VABS-II adaptive behavior, communication, socialization, and motor composite scores for ASD probands and toddler siblings with and without ASD. Score distributions overlap for probands and toddler siblings, with younger siblings exhibiting generally better performance in all domains. Statistics comparing proband and sibling performance among pairs concordant for ASD are reported in Additional file 1: Table S3 in the online supplemental material

Distributions of the VABS-II scores for probands and toddler siblings are depicted in Fig. 1, and a comparison of proband and sibling measures is presented in Additional file 1: Table S3. For scores on the VABS-II, significant ICCs (range 0.25–0.50) for concordant pairs were found for ABC, SOC, and COM composite scores and the EL and RL scale scores (Table 3). As expected, higher ICCs were observed in pairs concordant for ASD. The highest ICC was found for RL, where 66% of toddlers with ASD scored within 3 points (1 SD on the VABS-II scale scores) of their proband, as depicted in Additional file 1: Figure S2 in the online supplemental material. A significant ICC was also found between GM scores for discordant pairs (*r* = 0.18, *p* = 0.001), an association that did not survive correction for multiple comparisons in concordant pairs (*r* = 0.25, *p* = 0.053).

The results from regression models relating VABS-II scores among probands and siblings are shown in Table 4. Proband ABC, COM, EL, and RL were significantly associated with sibling scores in the same domains at 24 months, each surviving Bonferroni correction for effects of interest (proband score, proband score x group interaction) across models. No associations were found between proband and sibling SOC and GM scores after the adjustment for covariates. Significant proband score by sibling diagnostic group interactions was found for EL and RL scores, suggesting that associations between proband and sibling scores differed between concordant and discordant pairs, as expected. Highly similar main effects of proband scores (VABS-II ABC, EL, RL) were observed for the MSEL examiner-based assessments of general cognition (ELC), EL, and RL (Table 5).

**Table 4:** Linear regression analyses: VABS-II parent-reported behavior

	Beta^a^	95% CI lower	95% CI upper	*p* value
ABC model (*n* = 303)				
Proband ABC	0.33	0.11	0.55	0.004^+^
Sibling sex	− 0.27	− 0.48	− 0.07	0.009
Group	0.99	0.75	1.23	< 0.0001
Proband score x group	− 0.18	− 0.43	0.07	0.149
SOC model (*n* = 311)				
Proband SOC	0.25	0.04	0.46	0.022
Sibling sex	− 0.33	− 0.53	− 0.14	0.001
Group	0.88	0.64	1.12	< 0.0001
Proband score x group	− 0.21	− 0.45	0.03	0.089
COM model (*n* = 309)				
Proband COM	0.40	0.20	0.61	< 0.0001^+^
Sibling sex	− 0.16	− 0.35	0.04	0.113
Group	1.07	0.83	1.30	< 0.0001
Proband score x group	− 0.28	− 0.51	− 0.04	0.021
EL model (*n* = 317)				
Proband EL	0.44	0.23	0.65	< 0.0001^+^
Sibling sex	− 0.23	− 0.44	− 0.03	0.028
Group	0.80	0.55	1.04	< 0.0001
Proband score x group	− 0.36	− 0.60	− 0.12	0.003^+^
RL model (*n* = 318)				
Proband RL	0.53	0.32	0.73	< 0.0001^+^
Sibling sex	− 0.08	− 0.27	0.12	0.429
Group	1.05	0.82	1.28	< 0.0001
Proband score x group	− 0.42	− 0.65	− 0.19	< 0.0001^+^
GM model (*n* = 277)				
Proband GM	0.22	− 0.05	0.50	0.113
Sibling sex	0.03	− 0.22	0.27	0.832
Group	0.55	0.27	0.84	< 0.0001
Proband score x group	− 0.01	− 0.32	0.29	0.933

*ABC* Adaptive Behavior Composite, *SOC* socialization composite, *COM* communication composite, *EL* expressive language, *RL* receptive language, *GM* gross motor

^a^Standardized beta coefficients from linear regression models. Reference groups for sibling sex and group are female (vs. male) and ASD (vs. no ASD), respectively. Full model results are shown in Additional file 1: Table S4

^+^significant at *p* ≤ 0.004 after Bonferroni correction for main and interacting effects of proband score (12 comparisons)

**Table 5:** Linear regression analyses: MSEL examiner-based assessment

	Beta^a^	95% CI lower	95% CI upper	*p* value
ELC model (*n* = 317)				
Proband VABS-II ABC	0.30	0.09	0.50	0.004^+^
Sibling sex	− 0.29	− 0.49	− 0.10	0.003
Group	1.13	0.90	1.36	< 0.0001
Proband score x group	− 0.22	− 0.44	0.01	0.066
EL model (*n* = 324)				
Proband VABS-II EL	0.34	0.14	0.55	< 0.0001^+^
Sibling sex	− 0.18	− 0.38	0.03	0.089
Group	0.80	0.57	1.04	< 0.0001
Proband score x group	− 0.18	− 0.42	0.05	0.122
RL model (*n* = 323)				
Proband VABS-II RL	0.47	0.28	0.66	< 0.0001^+^
Sibling sex	− 0.14	− 0.33	0.05	0.140
Group	1.14	0.91	1.36	< 0.0001
Proband score x group	− 0.39	− 0.61	− 0.17	< 0.0001^+^

*ELC* Early Learning Composite, *EL* expressive language, *RL* receptive language

^a^Standardized beta coefficients from linear regression models. Reference groups for sibling sex and group are female (vs. male) and HR-ASD (vs. HR-NoASD), respectively. Full model results are shown in Additional file 1: Table S5

^+^significant at *p* ≤ 0.008 after Bonferroni correction for main and interacting effects of proband score (6 comparisons)

**Fig. 2:**
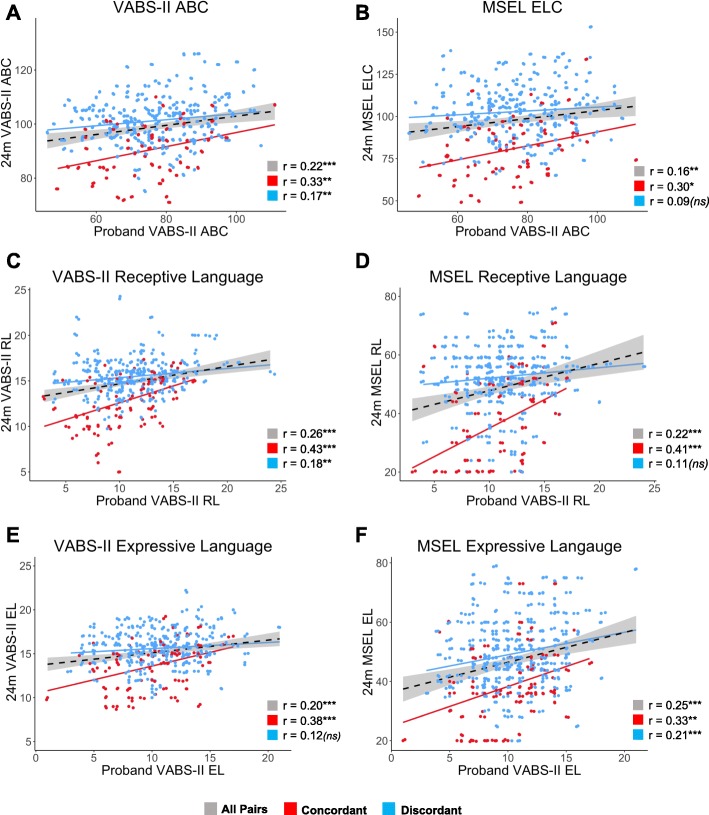
Raw scatterplots and bivariate Pearson correlations among proband and sibling scores. Plots are shown for all VABS-II and MSEL scores found to be significantly associated among proband-sibling pairs in regression analyses. The left panel depicts proband-sibling associations between identical domains on the VABS-II (**a**, **c**, **e**), while the right panel depicts associations between proband scores on the VABS-II and sibling scores on related domains from the MSEL (**b**, **d**, **f**). Overall linear associations for the entire sample (all probands, all siblings) are depicted by black dashed regression lines with shaded gray confidence intervals; corresponding correlations (computed for ease of effect size interpretation) are denoted by gray boxes. Linear associations and Pearson correlations for concordant pairs (red) and discordant pairs (blue) are also shown. Significance levels are denoted as follows: ns, non-significant; *p < 0.05, **p < 0.01, ***p < 0.001. Generally stronger associations are found for concordant pairs in all domains, with individuals with ASD exhibiting a downward shift in score profiles for adaptive behavior and cognition. Highly similar patterns of proband-sibling associations are found for overall functioning (**a**, **b**), receptive language (**c**, **d**), and expressive language (**e**, **f**) using both parent-reported VABS-II measures and MSEL examiner-based assessments of sibling abilities at 24 months

We found no notable associations between the sex of the proband, proband or sibling age, or study site and sibling outcomes at 24 months, and thus, these covariates are not presented in Tables 4 and 5; model results for the full set of covariates are reported in supplemental Additional file 1: Tables S4-S5. For the interpretation of the effect sizes, raw scatterplots and bivariate Pearson correlations among proband and sibling scores found to be significantly associated with the regression analyses on both the VABS-II and MSEL are shown in Fig. 2. Pearson correlations ranged between 0.16 and 0.26 for the entire sample, and between 0.30 and 0.43 for concordant pairs; thus, proband scores explained 9–18% of the variation (0.09 ≤ *r*^2^ ≤ 0.18) in adaptive behavior and communication in their toddler siblings with ASD.

All primary findings from ICCs and regression models relating proband and toddler sibling traits were highly similar across both the male-only sibling pairs and pairs where the proband scored > 60 on the VABS-II ABC (Additional file 1: Tables S6-S11). Models adjusted for maternal education level were highly similar to the main findings (Additional file 1: Tables S12–13). Replacing MSEL *T*-scores with age-adjusted scores had no impact on the results (Additional file 1: Table S14, Additional file 1: Figure S3).

## Discussion

The present study documents the associations between proband quantitative traits and toddler sibling diagnostic and dimensional outcomes at 24 months in a cohort of 385 proband-sibling pairs. We found that the proband ASD trait level, as measured by the SCQ, is predictive of recurrence risk in younger siblings, though ASD trait domains were not significantly correlated among concordant sibling pairs. Our analyses further revealed that proband adaptive behavior, communication, and expressive and receptive language scores accounted for significant variation in toddler sibling performance in the same domains, above and beyond major predictors of outcomes including diagnostic group. Associations were significant for the entire sample and stronger in concordant pairs, with proband scores explaining 9–18% of the variation in outcomes in toddler siblings diagnosed with ASD (0.30 ≤ *r* ≤ 0.43; Fig. 2). Importantly, these findings were convergent across both parent-reported and examiner-based assessments of sibling cognition and behavior at 24 months. This study demonstrates that ASD traits and cognitive and behavioral profiles in probands have the potential to identify the risk for recurrence and specific areas of developmental concern in younger siblings.

Given the heritable nature of ASD and elevated recurrence risk within families affected by ASD, we investigated whether proband traits were useful as statistical predictors of diagnostic outcomes in their younger siblings. The proband ASD trait level as measured by the SCQ significantly predicted sibling diagnostic outcome, such that a 1-point increase in the SCQ total score—reflective of endorsement of additional ASD symptoms—conferred a 6% increase in the odds of the later-born sibling receiving a diagnosis of ASD at 24 months. These findings are in contrast with two other high-risk infant sibling studies that did not find proband ASD traits to be predictive of recurrence [6, 16]. This may be due to the differences in the study design. In a similarly powered sample, Ozonoff and colleagues measured ASD traits in probands using the ADOS social communication score [6], which may not capture the same variability in ASD traits as the SCQ that also includes restricted and repetitive behaviors. The study by Schwichtenberg and colleagues [16] used the social responsiveness scale (SRS) to index ASD traits in probands and found that proband SRS was not predictive of categorical outcomes (ASD, atypical, typical) in younger siblings. However, that study [16] reported elevated ASD traits in multiple incidence (multiplex) families—as has been reported by others [33–35]—and found multiplex status (i.e., having more than one older child with ASD in the family) was a significant predictor of recurrence. These findings are consistent with the results from the current report, where elevated ASD traits were found in probands of siblings who developed ASD and are, by definition, multiplex families. Finally, we replicated previous findings that the sex of the sibling is a significant predictor of recurrence [6, 16] and that proband sex is not [6]. While additional studies will be needed to understand why certain indices of ASD traits in probands appear to be more predictive of recurrence in siblings than others, our findings suggest that indexing genetic liability for ASD in probands holds important information for identifying the risk for recurrence that deserves further investigation.

Although we identified that proband ASD trait level predicted recurrence risk in younger siblings, we found generally weak and non-significant associations between ASD trait domains (social interaction, communication, repetitive behaviors) in concordant pairs as measured by the ADI-R. Thus, while the syndrome itself is highly heritable, and elevated ASD traits travel in multiplex families where recurrence risk is highest, ASD symptomology appears to be phenotypically dissimilar among sibling pairs despite shared genetic background. Very similar weak associations have been reported in other studies of sibling pairs with ASD using the ADI-R [8, 11]. This may be reflective of a limitation of the ADI-R to index quantitative ASD traits, though cross-instrument correlations between proband SCQ and sibling ADOS scores were also weak and non-significant. Alternatively, it may indicate that ASD symptomology is influenced by non-shared environmental factors [8], as has been recently suggested by a study of twins phenotyped using the SRS [36] where twin-twin differences in SRS scores were notably greater above the diagnostic threshold for ASD. Finally, while it did not survive the correction for multiple comparisons, we did observe a notable association between qualitative abnormalities in the communication on the ADI-R—including lack of or delay in nonverbal gestures and social imitative play—among nonverbal sibling pairs concordant for ASD (Table 3), a finding that has been reported in twins with ASD and linked-to-shared genetic background [8, 14, 15]. This may suggest distinct patterns of association of ASD traits among siblings with and without comorbid intellectual disability that warrants further study.

Phenotypic congruence among siblings with ASD has been reported in areas outside of the diagnostic features of ASD including adaptive behavior, communication, socialization, and cognition [8–10, 12, 13]. In the present study, we extend these findings to a sample of 24-month-olds and their older siblings with ASD, demonstrating that global traits of adaptive behavior and communication are familial in nature and traceable to very early childhood. This is evidenced by a downward shift in the score distributions for ASD siblings and significant correlations between concordant proband-sibling pairs (Fig. 2) for adaptive behavior and communication that are in line with previous reports in biological siblings with ASD [8–10]. While measures of cognitive functioning in probands were not available, comparisons between proband adaptive behavior and toddler sibling general cognition revealed a significant positive association. Taken together, these findings highlight that proband adaptive behavior and communication abilities carry important information for sibling outcomes in the same domains at 24 months.

Proband-sibling associations were further investigated among domains of language development, a reported endophenotype of ASD [37]. Expressive and receptive languages were significantly correlated among sibling pairs, driven by pairs concordant for ASD (Table 3, Fig. 2). These results, importantly, were convergent across both parent-report and examiner-based assessments. Proband expressive language accounted for 14% of the variation in concordant sibling scores on the same VABS-II measure at 24 months (*r* = 0.38; Fig. 2). Receptive language associations were even stronger, with proband scores explaining the 18% of the variation in the scores of toddler siblings with ASD (*r* = 0.43; Fig. 2), and 66% of ASD siblings scoring within 1 SD of their proband (Additional file 1: Figure S3). These results echo findings that genetic liability for ASD impacts receptive language to a greater extent than expressive language [37]. This, to our knowledge, is the first evidence linking expressive and receptive language in sibling pairs concordant for ASD. Because language delay is observed by 12 months of age in infants who go on to receive an ASD diagnosis [17, 38] and occurs at greater frequency in high-risk siblings regardless of ASD diagnosis [37], this finding suggests that increased surveillance for language delays may be warranted in infant siblings of probands who exhibit marked deficits in expressive and receptive language.

Recent advances in individualized prediction algorithms in neuroscience are paving the way for identifying high-risk infants who will later be diagnosed with ASD as early as 6 months of age using neuroimaging [39, 40]. Another study has shown it is possible to predict dimensional cognitive abilities at age 2 from brain scans at birth in both typically developing children and preterm infants at risk for poor developmental outcomes [41]. This work is part of a larger shift in focus from the group to the individual [42], taking place in both research and practice, in keeping with the precision medicine framework designed to assign individuals to personal treatment plans, and in maximizing treatment efficacy [43]. It has been suggested that indices of genetic background, if shown to account for variation in child outcomes, may play a crucial role in the generation of neurodevelopmental risk algorithms capable of identifying individualized areas of concern [44], allowing for early, targeted intervention. Quantitative traits in first-degree relatives, as demonstrated in this study, may be particularly useful to include in such a prediction framework, especially in combination with other cost-effective measures that carry high predictive value for diagnostic outcome.

There is growing support for the hypothesis that ASD, which is both polygenic [3–5] and pleiotropic [45, 46] in nature, may be traceable to early-emerging developmental endophenotypes that are both specific and non-specific to ASD [47, 48]. This is evidenced by a body of work documenting that sensory, motor, and language behaviors are altered in the first year of life, prior to the onset of ASD symptoms [48, 49]. The need to explore genetic associations early, prior to symptom onset, is well illustrated in two recent twin studies. Hawks and colleagues [50] found that the variation in ASD traits and psychopathological traits non-specific to ASD were uncorrelated in infancy and traceable to genetically distinct structures, while these traits in childhood, after ASD develops, are largely overlapping, and thus conflating shared genetic influences with longitudinal, interactive effects. Pohl and colleagues [51] reported that highly heritable predictors of familial ASD recurrence—variation in attention, motor coordination, and parental ASD trait level—are also genetically independent in early childhood in the general population, yet jointly influence early reciprocal social behavior. Findings from the present report echo this work by demonstrating that it is ASD *endophenotypes* (language, adaptive behavior) and not ASD *traits* that are associated among concordant pairs. Taken together, this work emphasizes the importance of investigating the contribution of familial genetics to early precursor behavioral traits rather than to the diagnosis of ASD itself or to behaviors that emerge well after symptoms are evident [48].

Future work should focus on identifying how quantitative traits in both affected and unaffected family members, as indices of genetic liability for ASD and background genetic variation, relate to brain and behavioral development in infants through the period of risk to diagnosis. Such investigations will provide critical insights into how genetic liability for ASD influences neurodevelopmental and behavioral processes leading up to the onset of ASD symptomology, revealing mechanistic insights into pathogenesis [52]. Here, we demonstrate that proband adaptive behavior, communication, and language are associated with outcomes in those domains at 24 months in toddler siblings, but a developmental approach at multiple levels of analysis, including both brain and behavior, will be needed to understand the biological basis and temporal nature of these associations. Further, these studies should be extended to include more targeted behaviors, including eye tracking, for example, which has been shown to be highly heritable, disrupted in first-degree relatives, and aberrant in high-risk infants prior to diagnosis [53–56]. In the present study, motor skills were not associated among sibling pairs after adjustment for covariates, though there was an association among pairs for gross motor scores in the ICC analysis. This lack of significance may be due to the course nature of the motor assessments used in this study, as associations between more comprehensive motor assessments for twins concordant for ASD have been documented [57]. Future studies capturing more detailed measures of motor behaviors in proband-infant pairs would provide clarity.

### Limitations

There are certain limitations to the current study. The only measure of autistic features common to both probands and siblings was the ADI-R, which is a clinical measure not necessarily intended to capture continuous measures of severity across symptom domains. Further, the ADI-R is not well suited to capture the variability in ASD traits below the diagnostic threshold, and thus, there is a relatively little variability in the scores of the toddler siblings who did not develop ASD. Thus, conclusions related to a lack of association regarding autistic traits may be owed to measurement limitations, a common concern with other prior studies [8, 14, 15] that should be addressed in future investigations. Additionally, we did not have parental quantitative traits to provide a larger context for genetic background; future work is needed to understand the predictive utility of parental and proband quantitative traits for informing infant sibling outcomes. Measures of verbal and nonverbal intelligence in the probands were unavailable and limited our ability to fully characterize how phenotypic similarities in ASD traits among sibling pairs may vary as a function of similarities in intellectual ability. Finally, there is evidence that the number of siblings in a family with ASD (i.e., multiplex vs. simplex) is a strong predictor of outcomes in younger siblings; this information is currently being collected in the IBIS sample and will be explored in future analyses as an additional marker of the level of familial ASD genetic liability.

## Conclusions

The present study capitalized on the infant sibling study design to determine whether quantitative traits in probands were informative of outcomes in younger siblings. Here, we provide evidence that ASD traits in probands are predictive of recurrence risk and that quantitative traits in probands account for significant variation in sibling adaptive behavior, communication, and language abilities at 24 months. Our findings call for conducting deep phenotyping in first-degree relatives to parse the contributions of genetic background and genetic liability for ASD to brain and behavioral development in emerging ASD.

## Supplementary information

**Additional file 1: Figure S1**. ADI-R and SCQ score distributions. **Table S1**. Proband SCQ predicting sibling diagnostic outcome at 24-months. **Table S2**. Distribution of recurrence and predictors across SCQ quartiles. **Table S3.** Behavioral and clinical measures compared across concordant pairs. **Figure S2**. VABS-II RL score differences for concordant pairs. **Table S4.** Linear Regression Analyses: VABS-II Full Model Results. **Table S5**. Linear Regression Analyses: MSEL Full Model Results. **Table S6**. ICCs: Male-Only Sibling Pairs. **Table S7**. Linear regression analyses: VABS-II Parent Reported Behavior: Male-Only Pairs. **Table S8**. Linear regression analyses: MSEL Examiner-based Assessment: Male-Only Pairs. **Table S9**. ICCs: Pairs where Proband ABC > 60. **Table S10**. Linear regression analyses: VABS-II Parent Reported Behavior: Proband ABC >60. **Table S11**. Linear regression analyses: MSEL Examiner-based Assessment: Proband ABC >60. **Table S12**. Linear regression analyses: VABS-II Parent Reported Behavior: Maternal Education. **Table S13**. Linear regression analyses: MSEL Examiner-based Assessment: Maternal Education. **Table S14**. Linear regression analyses: MSEL Age-Adjusted Scores. **Figure S3**. Scatterplots: MSEL Age-Adjusted Scores.

## Data Availability

The datasets analyzed in the current study are available in the National Database for Autism Research (NDAR) repository in collection #19 titled “Longitudinal MRI Study of Infants at Risk for Autism”.

## Abbreviations

ABC: Adaptive Behavior Composite
ADI-R: Autism Diagnostic Interview-Revised
ADOS: Autism Diagnostic Observation Schedule
ANCOVA: Analysis of covariance
ASD: Autism spectrum disorder
COM: Communication
DSM-IV-TR: Diagnostic and Statistical Manual of Mental Disorders, Edition four, Text Revision
EL: Expressive language
ELC: Early Learning Composite
FIGS: Family Interview for Genetic Studies
FM: Fine motor
GM: Gross motor
IBIS: Infant Brain Imaging Study
ICC: Intraclass correlation
MS: Motor skills
MSEL: Mullen Scales of Early Learning
OR: Odds ratio
RL: Receptive language
SA: Social affect
SCQ: Social Communication Questionnaire
VABS-II: Vineland Adaptive Behavior Scales, Second Edition
