# Supplementary material for: Quantitative trait variation in ASD probands and toddler sibling outcomes at 24 months
Source: J Neurodev Disord. 2020 Feb 5;12:5. doi: 10.1186/s11689-020-9308-7 (PMC7003330; doi:10.1186/s11689-020-9308-7)
Supplement: Supplementary file 1 — Additional file 1: Figure S1. ADI-R and SCQ score distributions. Table S1. Proband SCQ predicting sibling diagnostic outcome at 24-months. Table S2. Distribution of recurrence and predictors across SCQ quartiles. Table S3. Behavioral and clinical measures compared across concordant pairs. Figure S2. VABS-II RL score differences for concordant pairs. Table S4. Linear Regression Analyses: VABS-II Full Model Results. Table S5. Linear Regression Analyses: MSEL Full Model Results. Table S6. ICCs: Male-Only Sibling Pairs. Table S7. Linear regression analyses: VABS-II Parent Reported Behavior: Male-Only Pairs. Table S8. Linear regression analyses: MSEL Examiner-based Assessment: Male-Only Pairs. Table S9. ICCs: Pairs where Proband ABC > 60. Table S10. Linear regression analyses: VABS-II Parent Reported Behavior: Proband ABC >60. Table S11. Linear regression analyses: MSEL Examiner-based Assessment: Proband ABC >60. Table S12. Linear regression analyses: VABS-II Parent Reported Behavior: Maternal Education. Table S13. Linear regression analyses: MSEL Examiner-based Assessment: Maternal Education. Table S14. Linear regression analyses: MSEL Age-Adjusted Scores. Figure S3. Scatterplots: MSEL Age-Adjusted Scores. [file 11689_2020_9308_MOESM1_ESM.docx]

**SUPPLEMENTAL RESULTS**

*Table of Contents:*

Figure S1 ADI-R and SCQ score distributions.

Table S1 Proband SCQ predicting sibling diagnostic outcome at 24-months

Table S2 Distribution of recurrence and predictors across SCQ quartiles

Table S3 Behavioral and clinical measures compared across concordant pairs

Figure S2 VABS-II RL score differences for concordant pairs

Table S4 Linear Regression Analyses: VABS-II Full Model Results

Table S5 Linear Regression Analyses: MSEL Full Model Results

Table S6 ICCs: Male-Only Sibling Pairs

Table S7 Linear regression analyses: VABS-II Parent Reported Behavior: Male-Only Pairs

Table S9 Linear regression analyses: MSEL Examiner-based Assessment: Male-Only Pairs

Table S9 ICCs: Pairs where Proband ABC > 60

Table S10 Linear regression analyses: VABS-II Parent Reported Behavior: Proband ABC >60

Table S11 Linear regression analyses: MSEL Examiner-based Assessment: Proband ABC >60

Table S12 Linear regression analyses: VABS-II Parent Reported Behavior: Maternal Education

Table S13 Linear regression analyses: MSEL Examiner-based Assessment: Maternal Education

Table S14 Linear regression analyses: MSEL Age-Adjusted Scores

Figure S3. Scatterplots: MSEL Age-Adjusted Scores

*Notes regarding linear models:* All models report standardized beta coefficients. Covariates include chronological age and sex of the proband and sibling, sibling diagnostic outcome group, and clinical study site. Reference groups for sex, sibling diagnostic group, and clinical study site are female (vs. male), ASD (vs. no ASD), and CHOP (Children’s hospital of Philadelphia) vs. all other clinical sites (UNC Chapel Hill, Washington University (STL), University of Washington in Seattle (SEA)), respectively.

**Figure S1. ADI-R and SCQ score distributions.** (A) Score distributions for proband SCQ split by probands of concordant (n = 89) and discordant (n = 296) pairs. (B-D) ADI-R score distributions for probands (top panels) and siblings (bottom panels) for the social, verbal communication, and non-verbal communication scores, respectively.

**Table S1. Proband SCQ predicting sibling diagnostic outcome at 24-months**

|  | Beta |  | SE |  | p-value |  | Odds  Ratio |  | 95% CI Lower |  | 95% CI Lower |
| --- | --- | --- | --- | --- | --- | --- | --- | --- | --- | --- | --- |
| **Model: SCQ Score^a^** |  |  |  |  |  |  |  |  |  |  |  |
| SCQ Score | 0.06 |  | 0.03 |  | 0.014 |  | 1.06 |  | 1.01 |  | 1.12 |
| Sibling Sex | 1.21 |  | 0.31 |  | 0.0001 |  | 3.34 |  | 1.84 |  | 6.36 |
| Proband Sex | -0.35 |  | 0.38 |  | 0.361 |  | 0.71 |  | 0.34 |  | 1.53 |
| Proband Age at SCQ | -0.009 |  | 0.01 |  | 0.056 |  | 0.99 |  | 0.47 |  | 2.04 |
| Site – SEA | -0.02 |  | 0.38 |  | 0.965 |  | 0.98 |  | 0.47 |  | 2.04 |
| Site – STL | -0.36 |  | 0.37 |  | 0.330 |  | 0.70 |  | 0.33 |  | 1.44 |
| Site – UNC | 0.04 |  | 0.39 |  | 0.911 |  | 1.04 |  | 0.48 |  | 2.23 |
| **Model: SCQ Quartile^b^** |  |  |  |  |  |  |  |  |  |  |  |
| SCQ Q2 | 0.60 |  | 0.43 |  | 0.162 |  | 1.81 |  | 0.79 |  | 4.27 |
| SCQ Q3 | 1.29 |  | 0.40 |  | 0.001 |  | 3.65 |  | 1.69 |  | 8.25 |
| SCQ Q4 | 0.84 |  | 0.44 |  | 0.054 |  | 2.31 |  | 1.00 |  | 5.55 |
| Sibling Sex | 1.18 |  | 0.32 |  | 0.0002 |  | 3.26 |  | 1.79 |  | 6.23 |
| Proband Sex | -0.37 |  | 0.39 |  | 0.333 |  | 0.69 |  | 0.33 |  | 1.50 |
| Proband Age at SCQ | -0.009 |  | 0.01 |  | 0.073 |  | 0.99 |  | 0.98 |  | 1.00 |
| Site – SEA | -0.08 |  | 0.38 |  | 0.831 |  | 0.92 |  | 0.44 |  | 1.94 |
| Site – STL | -0.44 |  | 0.38 |  | 0.239 |  | 0.64 |  | 0.30 |  | 1.34 |
| Site – UNC | 0.03 |  | 0.39 |  | 0.934 |  | 1.03 |  | 0.47 |  | 2.23 |
| **Model: SCQ Score + Verbal^c^** |  |  |  |  |  |  |  |  |  |  |  |
| SCQ Score | 0.06 |  | 0.02 |  | 0.024 |  | 1.06 |  | 1.01 |  | 1.11 |
| Proband verbal status | 0.56 |  | 0.30 |  | 0.066 |  | 1.75 |  | 0.96 |  | 3.16 |
| Sibling Sex | 1.18 |  | 0.31 |  | 0.0002 |  | 3.26 |  | 1.79 |  | 6.21 |
| Proband Sex | -0.32 |  | 0.38 |  | 0.399 |  | 0.72 |  | 0.35 |  | 1.58 |
| Proband Age at SCQ | -0.01 |  | 0.00 |  | 0.141 |  | 0.99 |  | 0.98 |  | 1.00 |
| Site – SEA | -0.09 |  | 0.38 |  | 0.811 |  | 0.91 |  | 0.43 |  | 1.92 |
| Site – STL | -0.42 |  | 0.37 |  | 0.262 |  | 0.66 |  | 0.31 |  | 1.37 |
| Site – UNC | -0.01 |  | 0.39 |  | 0.975 |  | 0.99 |  | 0.45 |  | 2.12 |
| **Model: SCQ Quartile + Verbal^d^** |  |  |  |  |  |  |  |  |  |  |  |
| SCQ Q2 | 0.53 |  | 0.43 |  | 0.221 |  | 1.69 |  | 0.73 |  | 4.01 |
| SCQ Q3 | 1.19 |  | 0.43 |  | 0.004 |  | 3.28 |  | 1.51 |  | 7.53 |
| SCQ Q4 | 0.80 |  | 0.41 |  | 0.068 |  | 2.22 |  | 0.95 |  | 5.35 |
| Proband verbal status | 0.46 |  | 0.31 |  | 0.138 |  | 1.59 |  | 0.85 |  | 2.90 |
| Sibling Sex | 1.16 |  | 0.32 |  | 0.0002 |  | 3.20 |  | 1.75 |  | 6.11 |
| Proband Sex | -0.34 |  | 0.39 |  | 0.377 |  | 0.71 |  | 0.34 |  | 1.56 |
| Proband Age at SCQ | -0.01 |  | 0.00 |  | 0.147 |  | 0.99 |  | 0.98 |  | 1.00 |
| Site – SEA | -0.13 |  | 0.38 |  | 0.726 |  | 0.87 |  | 0.41 |  | 1.85 |
| Site – STL | -0.48 |  | 0.38 |  | 0.202 |  | 0.62 |  | 0.29 |  | 1.29 |
| Site – UNC | -0.01 |  | 0.40 |  | 0.973 |  | 0.99 |  | 0.45 |  | 2.14 |
| ^a^Model includes SCQ as a continuous variable  ^b^Model includes proband SCQ quartile as a categorical predictor (Q1-Q4); Q1 is coded as the reference group.  ^c^Model includes SCQ score and verbal status of the proband; verbal is coded as the reference group.  ^d^Model includes SCQ quartile and verbal status of the proband; verbal is coded as the reference group. | | | | | | | | | | | |

**Table S2. Distribution of recurrence and predictors across SCQ quartiles**

|  | **Q1 (n = 90)**  SCQ [7,17] | |  | **Q2 (n = 85)**  SCQ [18,21] | |  | **Q3 (n = 92)**  SCQ [22,25] | |  | **Q4 (n = 79)**  SCQ [26,37] | |  | **ChiSq** |  | **p-val** |
| --- | --- | --- | --- | --- | --- | --- | --- | --- | --- | --- | --- | --- | --- | --- | --- |
|  | *N* | *%* |  | *N* | *%* |  | *N* | *%* |  | *N* | *%* |  |  |  |  |
| Sib ASD | 12 | **13%** |  | 17 | **20%** |  | 31 | **34%** |  | 18 | **23%** |  | 11.24 |  | 0.01 |
| Sib No-ASD | 78 | **87%** |  | 68 | **80%** |  | 61 | **66%** |  | 61 | **77%** |  |  |  |  |
|  |  |  |  |  |  |  |  |  |  |  |  |  |  |  |  |
| Sib Male | 51 | **57%** |  | 46 | **54%** |  | 64 | **70%** |  | 47 | **59%** |  | 5.16 |  | 0.16 |
| Sib Female | 39 | **43%** |  | 39 | **46%** |  | 28 | **30%** |  | 32 | **41%** |  |  |  |  |
|  |  |  |  |  |  |  |  |  |  |  |  |  |  |  |  |
| Pro Verbal | 77 | **86%** |  | 62 | **73%** |  | 60 | **65%** |  | 65 | **82%** |  | 12.64 |  | 0.006 |
| Pro NonVerbal | 13 | **14%** |  | 23 | **27%** |  | 32 | **35%** |  | 14 | **18%** |  |  |  |  |
| The pattern of recurrence risk across quartiles may be reflective of the significant difference in distribution of non-verbal probands, where the third quartile is comprised of nearly twice as many non-verbal probands as the fourth quartile, reflective of differences in the SCQ scoring algorithm for verbal and non-verbal children. | | | | | | | | | | | | | | | |

**Table S3. Behavioral and clinical measures compared across concordant siblings**

|  | **Probands** | |  | **Siblings** | |  | **Group Comparison** | | | |
| --- | --- | --- | --- | --- | --- | --- | --- | --- | --- | --- |
|  | *Mean* | *SD* |  | *Mean* | *SD* |  | *t* | *df* | *p* | *Cohen’s d* |
| **ADI-R** |  |  |  |  |  |  |  |  |  |  |
| Social | 19.02 | 5.69 |  | 8.98 | 5.36 |  | 12.35 | 166.1 | <0.001 | 1.90 |
| NV Communication | 11.35 | 2.22 |  | 6.47 | 3.63 |  | 8.90 | 91.67 | <0.001 | 1.62 |
| V Communication | 16.73 | 4.11 |  | 7.55 | 4.56 |  | 8.47 | 31.39 | <0.001 | 2.34 |
| **VABS-II** |  |  |  |  |  |  |  |  |  |  |
| ABC | 76.43 | 12.76 |  | 89.78 | 9.29 |  | -8.54 | 133.72 | <0.001 | -1.40 |
| Socialization | 71.31 | 12.57 |  | 89.59 | 9.30 |  | -10.28 | 139.98 | <0.001 | -1.66 |
| Communication | 80.39 | 16.79 |  | 89.05 | 11.44 |  | -4.95 | 134.94 | <0.001 | -0.80 |
| Expressive | 10.51 | 3.14 |  | 13.57 | 2.42 |  | -7.79 | 140.82 | <0.001 | -1.25 |
| Receptive | 11.19 | 3.16 |  | 12.77 | 2.59 |  | -4.75 | 142.53 | <0.001 | -0.76 |
| Motor | 83.07 | 13.35 |  | 95.09 | 8.51 |  | -8.23 | 129.95 | <0.001 | -1.37 |
| Fine | 12.13 | 2.88 |  | 14.51 | 1.51 |  | -8.14 | 110.43 | <0.001 | -1.38 |
| Gross | 12.37 | 2.18 |  | 14.33 | 1.78 |  | -6.08 | 137.67 | <0.001 | -1.01 |

**Figure S1. VABS-II RL score differences for concordant pairs**

Absolute differences in RL scores were calculated between probands and siblings concordant for ASD (n = 71). The majority of siblings scored very similarly to their proband, with 66% scoring within 1 SD (3 points).

**
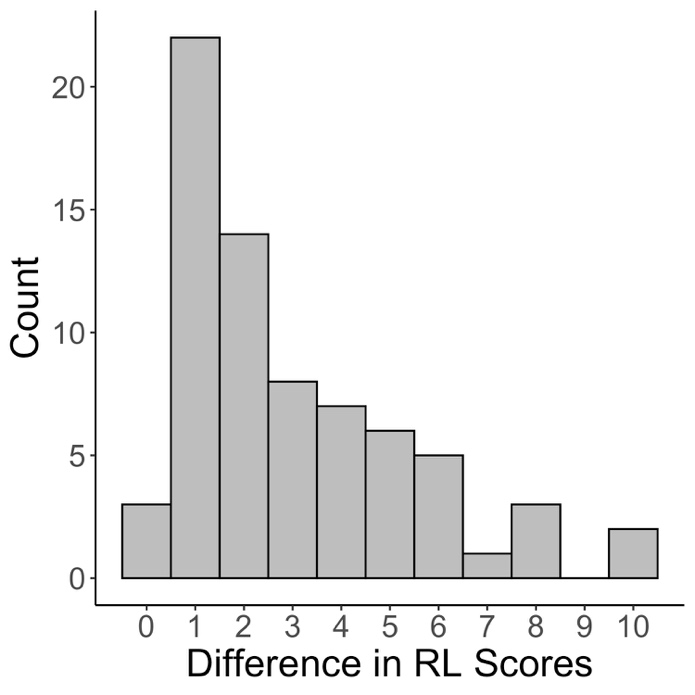
**

**Table S4. Linear Regression Analyses: VABS-II Full Model Results**

|  | **Standardized Beta** |  | **95% CI Lower** |  | **95% CI Upper** |  | **p-value** |
| --- | --- | --- | --- | --- | --- | --- | --- |
| **ABC Model** |  |  |  |  |  |  |  |
| Proband ABC | 0.33 |  | 0.11 |  | 0.55 |  | 0.004 |
| Sibling Sex | -0.27 |  | -0.48 |  | -0.07 |  | 0.009 |
| Proband Sex | -0.08 |  | -0.36 |  | 0.21 |  | 0.591 |
| Group | 0.99 |  | 0.75 |  | 1.23 |  | 0.000 |
| Proband Score x Group | -0.18 |  | -0.43 |  | 0.07 |  | 0.149 |
| Sibling Age | -0.09 |  | -0.19 |  | 0.02 |  | 0.110 |
| Proband Age | -0.02 |  | -0.12 |  | 0.08 |  | 0.666 |
| Site – SEA | -0.02 |  | -0.30 |  | 0.27 |  | 0.914 |
| Site – STL | -0.12 |  | -0.37 |  | 0.14 |  | 0.366 |
| Site – UNC | -0.16 |  | -0.44 |  | 0.13 |  | 0.282 |
| **SOC Model** |  |  |  |  |  |  |  |
| Proband SOC | 0.25 |  | 0.04 |  | 0.46 |  | 0.022 |
| Sibling Sex | -0.33 |  | -0.53 |  | -0.14 |  | 0.001 |
| Proband Sex | -0.19 |  | -0.47 |  | 0.10 |  | 0.194 |
| Group | 0.88 |  | 0.64 |  | 1.12 |  | 0.000 |
| Proband Score x Group | -0.21 |  | -0.45 |  | 0.03 |  | 0.089 |
| Sibling Age | -0.05 |  | -0.15 |  | 0.05 |  | 0.325 |
| Proband Age | -0.07 |  | -0.17 |  | 0.03 |  | 0.148 |
| Site – SEA | -0.10 |  | -0.38 |  | 0.18 |  | 0.487 |
| Site – STL | 0.19 |  | -0.06 |  | 0.44 |  | 0.133 |
| Site – UNC | -0.48 |  | -0.77 |  | -0.20 |  | 0.000 |
| **COM Model** |  |  |  |  |  |  |  |
| Proband COM | 0.40 |  | 0.20 |  | 0.61 |  | 0.000 |
| Sibling Sex | -0.16 |  | -0.35 |  | 0.04 |  | 0.113 |
| Proband Sex | -0.02 |  | -0.29 |  | 0.26 |  | 0.899 |
| Group | 1.07 |  | 0.83 |  | 1.30 |  | 0.000 |
| Proband Score x Group | -0.28 |  | -0.51 |  | -0.04 |  | 0.021 |
| Sibling Age | -0.04 |  | -0.14 |  | 0.06 |  | 0.459 |
| Proband Age | -0.06 |  | -0.15 |  | 0.04 |  | 0.219 |
| Site – SEA | -0.02 |  | -0.29 |  | 0.26 |  | 0.913 |
| Site – STL | -0.17 |  | -0.42 |  | 0.08 |  | 0.173 |
| Site – UNC | -0.24 |  | -0.52 |  | 0.03 |  | 0.085 |
| **EL Model** |  |  |  |  |  |  |  |
| Proband EL | 0.44 |  | 0.23 |  | 0.65 |  | 0.000 |
| Sibling Sex | -0.23 |  | -0.44 |  | -0.03 |  | 0.028 |
| Proband Sex | -0.05 |  | -0.34 |  | 0.24 |  | 0.716 |
| Group | 0.80 |  | 0.55 |  | 1.04 |  | 0.000 |
| Proband Score x Group | -0.36 |  | -0.60 |  | -0.12 |  | 0.003 |
| Sibling Age | -0.06 |  | -0.17 |  | 0.04 |  | 0.229 |
| Proband Age | -0.04 |  | -0.14 |  | 0.06 |  | 0.483 |
| Site – SEA | -0.17 |  | -0.46 |  | 0.11 |  | 0.238 |
| Site – STL | -0.24 |  | -0.51 |  | 0.02 |  | 0.073 |
| Site – UNC | -0.30 |  | -0.60 |  | 0.00 |  | 0.049 |
| **RL Model** |  |  |  |  |  |  |  |
| Proband RL | 0.53 |  | 0.32 |  | 0.73 |  | 0.000 |
| Sibling Sex | -0.08 |  | -0.27 |  | 0.12 |  | 0.429 |
| Proband Sex | 0.08 |  | -0.19 |  | 0.36 |  | 0.557 |
| Group | 1.05 |  | 0.82 |  | 1.28 |  | 0.000 |
| Proband Score x Group | -0.42 |  | -0.65 |  | -0.19 |  | 0.000 |
| Sibling Age | -0.05 |  | -0.14 |  | 0.05 |  | 0.342 |
| Proband Age | -0.05 |  | -0.14 |  | 0.04 |  | 0.302 |
| Site – SEA | 0.07 |  | -0.19 |  | 0.34 |  | 0.588 |
| Site – STL | -0.08 |  | -0.32 |  | 0.17 |  | 0.541 |
| Site – UNC | -0.26 |  | -0.54 |  | 0.02 |  | 0.065 |
| **GM Model** |  |  |  |  |  |  |  |
| Proband GM | 0.22 |  | -0.05 |  | 0.50 |  | 0.113 |
| Sibling Sex | 0.03 |  | -0.22 |  | 0.27 |  | 0.832 |
| Proband Sex | 0.05 |  | -0.29 |  | 0.38 |  | 0.792 |
| Group | 0.55 |  | 0.27 |  | 0.84 |  | 0.000 |
| Proband Score x Group | -0.01 |  | -0.32 |  | 0.29 |  | 0.933 |
| Sibling Age | -0.13 |  | -0.25 |  | -0.02 |  | 0.024 |
| Proband Age | 0.11 |  | -0.03 |  | 0.24 |  | 0.124 |
| Site – SEA | -0.15 |  | -0.50 |  | 0.20 |  | 0.413 |
| Site – STL | -0.24 |  | -0.55 |  | 0.07 |  | 0.133 |
| Site – UNC | 0.02 |  | -0.32 |  | 0.36 |  | 0.917 |

**Table S5. Linear Regression Analyses: MSEL Full Model Results**

|  | **Standardized Beta** |  | **95% CI Lower** |  | **95% CI Upper** |  | **p-value** |
| --- | --- | --- | --- | --- | --- | --- | --- |
| **ELC Model** | 0.30 |  | 0.09 |  | 0.50 |  | 0.004 |
| Proband ELC | -0.29 |  | -0.49 |  | -0.10 |  | 0.003 |
| Sibling Sex | 0.06 |  | -0.22 |  | 0.33 |  | 0.689 |
| Proband Sex | 1.13 |  | 0.90 |  | 1.36 |  | 0.000 |
| Group | -0.22 |  | -0.44 |  | 0.01 |  | 0.066 |
| Proband Score x Group | -0.03 |  | -0.13 |  | 0.06 |  | 0.472 |
| Sibling Age | -0.04 |  | -0.13 |  | 0.05 |  | 0.389 |
| Proband Age | 0.08 |  | -0.19 |  | 0.35 |  | 0.566 |
| Site – SEA | -0.12 |  | -0.36 |  | 0.13 |  | 0.347 |
| Site – STL | 0.24 |  | -0.04 |  | 0.52 |  | 0.091 |
| Site – UNC | 0.30 |  | 0.09 |  | 0.50 |  | 0.004 |
| **EL Model** |  |  |  |  |  |  |  |
| Proband EL | 0.34 |  | 0.14 |  | 0.55 |  | 0.001 |
| Sibling Sex | -0.18 |  | -0.38 |  | 0.03 |  | 0.089 |
| Proband Sex | -0.07 |  | -0.36 |  | 0.21 |  | 0.625 |
| Group | 0.80 |  | 0.57 |  | 1.04 |  | 0.000 |
| Proband Score x Group | -0.18 |  | -0.42 |  | 0.05 |  | 0.122 |
| Sibling Age | -0.01 |  | -0.11 |  | 0.09 |  | 0.839 |
| Proband Age | -0.05 |  | -0.15 |  | 0.05 |  | 0.317 |
| Site – SEA | -0.30 |  | -0.58 |  | -0.02 |  | 0.034 |
| Site – STL | -0.22 |  | -0.47 |  | 0.04 |  | 0.099 |
| Site – UNC | 0.21 |  | -0.08 |  | 0.50 |  | 0.148 |
| **RL Model** |  |  |  |  |  |  |  |
| Proband RL | 0.47 |  | 0.28 |  | 0.66 |  | 0.000 |
| Sibling Sex | -0.14 |  | -0.33 |  | 0.05 |  | 0.140 |
| Proband Sex | 0.00 |  | -0.27 |  | 0.27 |  | 0.990 |
| Group | 1.14 |  | 0.91 |  | 1.36 |  | 0.000 |
| Proband Score x Group | -0.39 |  | -0.61 |  | -0.17 |  | 0.000 |
| Sibling Age | -0.06 |  | -0.16 |  | 0.03 |  | 0.179 |
| Proband Age | -0.09 |  | -0.18 |  | 0.00 |  | 0.061 |
| Site – SEA | 0.09 |  | -0.17 |  | 0.36 |  | 0.500 |
| Site – STL | 0.02 |  | -0.22 |  | 0.26 |  | 0.859 |
| Site – UNC | -0.01 |  | -0.28 |  | 0.26 |  | 0.934 |

**Table S6. ICCs: Male-Only Sibling Pairs**

|  |  | **Concordant**  **Pairs** | | |  | **Discordant**  **Pairs** | | |
| --- | --- | --- | --- | --- | --- | --- | --- | --- |
|  |  | *n* | *ICC* | *p* |  | *n* | *ICC* | *p* |
| **ADI-R** |  |  |  |  |  |  |  |  |
| Social |  | 58 | 0.01 | 0.441 |  | 134 | -0.01 | 0.631 |
| RRB |  | 58 | 0.02 | 0.402 |  | 134 | 0.02 | 0.284 |
| NV Communication |  | 21 | 0.12 | 0.262 |  | 16 | 0.09 | 0.097 |
| V Communication |  | 10 | 0.10 | 0.172 |  | 41 | -0.03 | 0.776 |
| **VABS-II** |  |  |  |  |  |  |  |  |
| ABC |  | 47 | 0.26 | 0.016 |  | 117 | 0.10 | 0.028 |
| Socialization |  | 55 | 0.25 | 0.007 |  | 118 | 0.01 | 0.463 |
| Communication |  | 49 | 0.35 | 0.018 |  | 118 | 0.11 | 0.087 |
| Expressive |  | 51 | 0.30 | 0.011 |  | 119 | 0.01 | 0.405 |
| Receptive |  | 51 | 0.50 | 0.001^+^ |  | 120 | 0.16 | 0.017 |
| Motor |  | 44 | -0.16 | 0.834 |  | 105 | 0.18 | 0.032 |
| Fine |  | 43 | -0.16 | 0.851 |  | 106 | 0.10 | 0.202 |
| Gross |  | 43 | 0.32 | 0.040 |  | 105 | 0.28 | <0.001^+^ |
| **^+^**significant at p ≤ 0.004 after Bonferroni correction (12 comparisons). | | | | | | | | |

**Table S7. Linear regression analyses: VABS-II Parent Reported Behavior: Male-Only Pairs**

|  | Beta^a^ |  | 95% CI Lower |  | 95% CI Upper |  | p-value |
| --- | --- | --- | --- | --- | --- | --- | --- |
| **ABC Model** |  |  |  |  |  |  |  |
| Proband ABC | 0.37 |  | 0.12 |  | 0.63 |  | 0.005**^+^** |
| Group | 0.94 |  | 0.64 |  | 1.24 |  | <0.0001 |
| Proband Score x Group | -0.22 |  | -0.52 |  | 0.09 |  | 0.161 |
|  |  |  |  |  |  |  |  |
| **SOC Model** |  |  |  |  |  |  |  |
| Proband SOC | 0.41 |  | 0.16 |  | 0.67 |  | 0.002**^+^** |
| Group | 0.78 |  | 0.46 |  | 1.06 |  | <0.0001 |
| Proband Score x Group | -0.43 |  | -0.73 |  | -0.12 |  | 0.006 |
|  |  |  |  |  |  |  |  |
| **COM Model** |  |  |  |  |  |  |  |
| Proband COM | 0.37 |  | 0.12 |  | 0.63 |  | 0.004**^+^** |
| Group | 1.02 |  | 0.72 |  | 1.32 |  | <0.0001 |
| Proband Score x Group | -0.28 |  | -0.58 |  | 0.02 |  | 0.069 |
|  |  |  |  |  |  |  |  |
| **EL Model** |  |  |  |  |  |  |  |
| Proband EL | 0.36 |  | 0.12 |  | 0.60 |  | 0.003**^+^** |
| Group | 0.86 |  | 0.56 |  | 1.16 |  | <0.0001 |
| Proband Score x Group | -0.38 |  | -0.68 |  | -0.08 |  | 0.014 |
|  |  |  |  |  |  |  |  |
| **RL Model** |  |  |  |  |  |  |  |
| Proband RL | 0.57 |  | 0.33 |  | 0.82 |  | <0.0001**^+^** |
| Group | 0.91 |  | 0.63 |  | 1.20 |  | <0.0001 |
| Proband Score x Group | -0.47 |  | -0.76 |  | -0.18 |  | 0.002**^+^** |
|  |  |  |  |  |  |  |  |
| **GM Model** |  |  |  |  |  |  |  |
| Proband GM | 0.28 |  | -0.04 |  | 0.60 |  | 0.087 |
| Group | 0.63 |  | 0.30 |  | 0.96 |  | <0.0001 |
| Proband Score x Group | -0.02 |  | -0.38 |  | 0.34 |  | 0.899 |
|  |  |  |  |  |  |  |  |
| *ABC* Adaptive Behavior Composite, *SOC* Socialization composite, *COM* Communication composite,  *EL* Expressive Language*, RL* Receptive Language*, GM* Gross Motor  ^a^Standardized beta coefficients from linear regression models. Reference groups for Sibling sex and Group are female (vs. male) and ASD (vs. no ASD), respectively.  **^+^**significant at p ≤ 0.004 after Bonferroni correction for main and interacting  effects of proband score (12 comparisons). | | | | | | | |

**Table S8. Linear regression analyses: MSEL Examiner-based Assessment: Male-Only Pairs**

|  | Beta^a^ |  | 95% CI Lower |  | 95% CI Upper |  | p-value |
| --- | --- | --- | --- | --- | --- | --- | --- |
| **ELC Model** |  |  |  |  |  |  |  |
| Proband VABS-II ABC | 0.27 |  | 0.04 |  | 0.51 |  | 0.025 |
| Group | 1.10 |  | 0.81 |  | 1.39 |  | <0.0001 |
| Proband Score x Group | -0.25 |  | -0.54 |  | 0.04 |  | 0.086 |
|  |  |  |  |  |  |  |  |
| **EL Model** |  |  |  |  |  |  |  |
| Proband VABS-II EL | 0.32 |  | 0.08 |  | 0.56 |  | 0.010 |
| Group | 0.72 |  | 0.41 |  | 1.02 |  | <0.0001 |
| Proband Score x Group | -0.23 |  | -0.53 |  | 0.07 |  | 0.132 |
|  |  |  |  |  |  |  |  |
| **RL Model** |  |  |  |  |  |  |  |
| Proband VABS-II RL | 0.42 |  | 0.19 |  | 0.66 |  | <0.0001**^+^** |
| Group | 1.08 |  | 0.80 |  | 1.37 |  | <0.0001 |
| Proband Score x Group | -0.41 |  | -0.69 |  | -0.13 |  | 0.005**^+^** |
|  |  |  |  |  |  |  |  |
| *ELC* Early Learning Composite, *EL* Expressive Language*, RL* Receptive Language  ^a^Standardized beta coefficients from linear regression models. Reference groups for Sibling sex and Group are female (vs. male) and HR-ASD (vs. HR-NoASD), respectively.  **^+^**significant at p ≤ 0.008 after Bonferroni correction for main and interacting  effects of proband score (6 comparisons). | | | | | | | |

**Table S9. ICCs: Pairs where Proband ABC > 60**

|  |  | **Concordant**  **Pairs** | | |  | **Discordant**  **Pairs** | | |
| --- | --- | --- | --- | --- | --- | --- | --- | --- |
|  |  | *n* | *ICC* | *p* |  | *n* | *ICC* | *p* |
| **ADI-R** |  |  |  |  |  |  |  |  |
| Social |  | 58 | -0.10 | 0.787 |  | 211 | 0.04 | 0.042 |
| RRB |  | 58 | 0.00 | 0.483 |  | 211 | 0.04 | 0.074 |
| NV Communication |  | 16 | 0.40 | 0.030 |  | 21 | 0.05 | 0.209 |
| V Communication |  | 18 | 0.08 | 0.209 |  | 83 | 0.01 | 0.369 |
| **VABS-II** |  |  |  |  |  |  |  |  |
| ABC |  | 56 | 0.24 | 0.022 |  | 215 | 0.11 | 0.002**^+^** |
| Socialization |  | 56 | 0.26 | 0.007 |  | 216 | 0.05 | 0.092 |
| Communication |  | 56 | 0.42 | 0.008 |  | 223 | 0.20 | <0.001**^+^** |
| Expressive |  | 58 | 0.41 | 0.001**^+^** |  | 219 | 0.09 | 0.026 |
| Receptive |  | 59 | 0.51 | 0.001**^+^** |  | 220 | 0.18 | 0.001**^+^** |
| Motor |  | 51 | 0.10 | 0.258 |  | 197 | 0.12 | 0.058 |
| Fine |  | 51 | -0.04 | 0.587 |  | 197 | 0.07 | 0.228 |
| Gross |  | 51 | 0.31 | 0.029 |  | 196 | 0.22 | <0.001**^+^** |
| **^+^**significant at p ≤ 0.004 after Bonferroni correction (12 comparisons). | | | | | | | | |

**Table S10. Linear regression analyses: VABS-II Parent Reported Behavior: Proband ABC >60**

|  | Beta^a^ |  | 95% CI Lower |  | 95% CI Upper |  | p-value |
| --- | --- | --- | --- | --- | --- | --- | --- |
| **ABC Model** |  |  |  |  |  |  |  |
| Proband ABC | 0.30 |  | 0.05 |  | 0.54 |  | 0.017 |
| Group | 0.92 |  | 0.65 |  | 1.19 |  | <0.0001 |
| Proband Score x Group | -0.11 |  | -0.38 |  | 0.16 |  | 0.412 |
|  |  |  |  |  |  |  |  |
| **SOC Model** |  |  |  |  |  |  |  |
| Proband SOC | 0.27 |  | 0.02 |  | 0.51 |  | 0.032 |
| Group | 0.82 |  | 0.56 |  | 1.09 |  | <0.0001 |
| Proband Score x Group | -0.21 |  | -0.48 |  | 0.06 |  | 0.132 |
|  |  |  |  |  |  |  |  |
| **COM Model** |  |  |  |  |  |  |  |
| Proband COM | 0.40 |  | 0.15 |  | 0.64 |  | 0.002**^+^** |
| Group | 1.01 |  | 0.75 |  | 1.27 |  | <0.0001 |
| Proband Score x Group | -0.23 |  | -0.50 |  | 0.04 |  | 0.097 |
|  |  |  |  |  |  |  |  |
| **EL Model** |  |  |  |  |  |  |  |
| Proband EL | 0.53 |  | 0.27 |  | 0.78 |  | <0.0001**^+^** |
| Group | 0.74 |  | 0.47 |  | 1.01 |  | <0.0001 |
| Proband Score x Group | -0.44 |  | -0.72 |  | -0.16 |  | 0.002**^+^** |
|  |  |  |  |  |  |  |  |
| **RL Model** |  |  |  |  |  |  |  |
| Proband RL | 0.50 |  | 0.27 |  | 0.73 |  | <0.0001**^+^** |
| Group | 0.99 |  | 0.73 |  | 1.24 |  | <0.0001 |
| Proband Score x Group | -0.35 |  | -0.60 |  | -0.09 |  | 0.008 |
|  |  |  |  |  |  |  |  |
| **GM Model** |  |  |  |  |  |  |  |
| Proband GM | 0.25 |  | -0.02 |  | 0.52 |  | 0.073 |
| Group | 0.46 |  | 0.17 |  | 0.76 |  | 0.002 |
| Proband Score x Group | -0.03 |  | -0.33 |  | 0.27 |  | 0.858 |
|  |  |  |  |  |  |  |  |
| *ABC* Adaptive Behavior Composite, *SOC* Socialization composite, *COM* Communication composite,  *EL* Expressive Language*, RL* Receptive Language*, GM* Gross Motor  ^a^Standardized beta coefficients from linear regression models. Reference groups for Sibling sex and Group are female (vs. male) and ASD (vs. no ASD), respectively.  **^+^**significant at p ≤ 0.004 after Bonferroni correction for main and interacting  effects of proband score (12 comparisons). | | | | | | | |

**Table S11. Linear regression analyses: MSEL Examiner-based Assessment: Proband ABC >60**

|  | Beta^a^ |  | 95% CI Lower |  | 95% CI Upper |  | p-value |
| --- | --- | --- | --- | --- | --- | --- | --- |
| **ELC Model** |  |  |  |  |  |  |  |
| Proband VABS-II ABC | 0.25 |  | 0.04 |  | 0.47 |  | 0.023 |
| Group | 1.07 |  | 0.82 |  | 1.32 |  | <0.0001 |
| Proband Score x Group | -0.13 |  | -0.37 |  | 0.12 |  | 0.315 |
|  |  |  |  |  |  |  |  |
| **EL Model** |  |  |  |  |  |  |  |
| Proband VABS-II EL | 0.51 |  | 0.27 |  | 0.75 |  | <0.0001**^+^** |
| Group | 0.81 |  | 0.55 |  | 1.06 |  | <0.0001 |
| Proband Score x Group | -0.35 |  | -0.61 |  | -0.08 |  | 0.011 |
|  |  |  |  |  |  |  |  |
| **RL Model** |  |  |  |  |  |  |  |
| Proband VABS-II RL | 0.47 |  | 0.26 |  | 0.68 |  | <0.0001**^+^** |
| Group | 1.06 |  | 0.80 |  | 1.31 |  | <0.0001 |
| Proband Score x Group | -0.35 |  | -0.59 |  | -0.11 |  | 0.004**^+^** |
|  |  |  |  |  |  |  |  |
| *ELC* Early Learning Composite, *EL* Expressive Language*, RL* Receptive Language  ^a^Standardized beta coefficients from linear regression models. Reference groups for Sibling sex and Group are female (vs. male) and HR-ASD (vs. HR-NoASD), respectively.  **^+^**significant at p ≤ 0.008 after Bonferroni correction for main and interacting  effects of proband score (6 comparisons). | | | | | | | |

**Table S12. Linear regression analyses: VABS-II Parent Reported Behavior: Maternal Education**

|  | **Standardized Beta** |  | **95% CI Lower** |  | **95% CI Upper** |  | **p-value** |
| --- | --- | --- | --- | --- | --- | --- | --- |
| **ABC Model** |  |  |  |  |  |  |  |
| Proband ABC | 0.31 |  | 0.09 |  | 0.54 |  | 0.006 |
| Group | 1.00 |  | 0.76 |  | 1.25 |  | <0.0001 |
| Proband Score x Group | -0.18 |  | -0.43 |  | 0.07 |  | 0.156 |
| MEDU – College^b^ | 0.17 |  | -0.06 |  | 0.40 |  | 0.153 |
| MEDU – Grad Degree | 0.22 |  | -0.05 |  | 0.49 |  | 0.113 |
| **SOC Model** |  |  |  |  |  |  |  |
| Proband SOC | 0.25 |  | 0.03 |  | 0.46 |  | 0.023 |
| Group | 0.88 |  | 0.64 |  | 1.12 |  | <0.0001 |
| Proband Score x Group | -0.22 |  | -0.46 |  | 0.03 |  | 0.079 |
| MEDU – College | 0.16 |  | -0.07 |  | 0.39 |  | 0.164 |
| MEDU – Grad Degree | 0.15 |  | -0.12 |  | 0.42 |  | 0.270 |
| **COM Model** |  |  |  |  |  |  |  |
| Proband COM | 0.37 |  | 0.16 |  | 0.57 |  | 0.001**^+^** |
| Group | 0.08 |  | -0.20 |  | 0.36 |  | 0.565 |
| Proband Score x Group | -0.13 |  | -0.38 |  | 0.12 |  | 0.312 |
| MEDU – College | 0.25 |  | 0.03 |  | 0.47 |  | 0.026 |
| MEDU – Grad Degree | 0.32 |  | 0.06 |  | 0.58 |  | 0.017 |
| **EL Model** |  |  |  |  |  |  |  |
| Proband EL | 0.43 |  | 0.21 |  | 0.64 |  | <0.0001**^+^** |
| Group | 0.84 |  | 0.59 |  | 1.08 |  | <0.0001 |
| Proband Score x Group | -0.33 |  | -0.57 |  | -0.09 |  | 0.007 |
| MEDU – College | 0.28 |  | 0.05 |  | 0.51 |  | 0.018 |
| MEDU – Grad Degree | 0.33 |  | 0.06 |  | 0.60 |  | 0.017 |
| **RL Model** |  |  |  |  |  |  |  |
| Proband RL | 0.48 |  | 0.28 |  | 0.69 |  | <0.0001**^+^** |
| Group | 1.05 |  | 0.82 |  | 1.29 |  | <0.0001 |
| Proband Score x Group | -0.40 |  | -0.63 |  | -0.16 |  | 0.001**^+^** |
| MEDU – College | 0.19 |  | -0.03 |  | 0.41 |  | 0.091 |
| MEDU – Grad Degree | 0.27 |  | 0.01 |  | 0.53 |  | 0.042 |
| **GM Model** |  |  |  |  |  |  |  |
| Proband GM | 0.24 |  | -0.03 |  | 0.51 |  | 0.085 |
| Group | 0.60 |  | 0.32 |  | 0.89 |  | <0.0001 |
| Proband Score x Group | -0.06 |  | -0.36 |  | 0.24 |  | 0.689 |
| MEDU – College | 0.03 |  | -0.25 |  | 0.30 |  | 0.846 |
| MEDU – Grad Degree | 0.06 |  | -0.26 |  | 0.38 |  | 0.718 |
| *ABC* Adaptive Behavior Composite, *COM* Communication composite, *SOC* Socialization composite *EL* Expressive Language*, RL* Receptive Language*, GM* Gross Motor  ^a^Standardized beta coefficients from linear regression models. Reference groups for Sibling sex and Group are female (vs. male) and HR-ASD (vs. HR-NoASD), respectively.  ^b^Maternal education coded as a 3-level variable: high school (reference group), college degree (College), graduate degree (Grad Degree).  **^+^**significant at p ≤ 0.004 after Bonferroni correction for main and interacting effects of proband score (12 comparisons). | | | | | | | |

**Table S13. Linear regression analyses: MSEL Assessment: Maternal Education**

|  | **Standardized Beta** |  | **95% CI Lower** |  | **95% CI Upper** |  | **p-value** |
| --- | --- | --- | --- | --- | --- | --- | --- |
| **ELC Model** |  |  |  |  |  |  |  |
| Proband ABC | 0.27 |  | 0.06 |  | 0.47 |  | 0.011 |
| Group | 1.13 |  | 0.90 |  | 1.36 |  | <0.0001 |
| Proband Score x Group | -0.20 |  | -0.43 |  | 0.03 |  | 0.095 |
| MEDU – College^b^ | 0.29 |  | 0.07 |  | 0.50 |  | 0.010 |
| MEDU – Grad Degree | 0.35 |  | 0.09 |  | 0.60 |  | 0.008 |
| **EL Model** |  |  |  |  |  |  |  |
| Proband EL | 0.32 |  | 0.11 |  | 0.53 |  | 0.002**^+^** |
| Group | 0.81 |  | 0.58 |  | 1.04 |  | <0.0001 |
| Proband Score x Group | -0.16 |  | -0.39 |  | 0.08 |  | 0.187 |
| MEDU – College | 0.31 |  | 0.08 |  | 0.53 |  | 0.007 |
| MEDU – Grad Degree | 0.30 |  | 0.04 |  | 0.56 |  | 0.026 |
| **RL Model** |  |  |  |  |  |  |  |
| Proband RL | 0.45 |  | 0.26 |  | 0.65 |  | <0.0001**^+^** |
| Group | 1.14 |  | 0.92 |  | 1.37 |  | <0.0001 |
| Proband Score x Group | -0.40 |  | -0.62 |  | -0.18 |  | <0.0001**^+^** |
| MEDU – College | 0.26 |  | 0.04 |  | 0.47 |  | 0.018 |
| MEDU – Grad Degree | 0.30 |  | 0.05 |  | 0.55 |  | 0.021 |
| *ELC* Early Learning Composite, *EL* Expressive Language*, RL* Receptive Language  ^a^Standardized beta coefficients from linear regression models. Reference groups for Sibling sex and Group are female (vs. male) and HR-ASD (vs. HR-NoASD), respectively.  ^b^Maternal education coded as a 3-level variable: high school (reference group), college degree (College), graduate degree (Grad Degree).  **^+^**significant at p ≤ 0.008 after Bonferroni correction for main and interacting effects of proband score (6 comparisons). | | | | | | | |

**Table S14. Linear regression analyses: MSEL Age-Adjusted Scores**

|  | Beta^a^ |  | 95% CI Lower |  | 95% CI Upper |  | p-value |
| --- | --- | --- | --- | --- | --- | --- | --- |
| **EL Model** |  |  |  |  |  |  |  |
| Proband VABS-II EL | 0.36 |  | 0.15 |  | 0.56 |  | <0.0001**^+^** |
| Group | 0.79 |  | 0.55 |  | 1.02 |  | <0.0001 |
| Proband Score x Group | -0.21 |  | -0.44 |  | 0.03 |  | 0.081 |
|  |  |  |  |  |  |  |  |
| **RL Model** |  |  |  |  |  |  |  |
| Proband VABS-II RL | 0.50 |  | 0.31 |  | 0.68 |  | <0.0001**^+^** |
| Group | 1.16 |  | 0.94 |  | 1.38 |  | <0.0001 |
| Proband Score x Group | -0.43 |  | -0.65 |  | -0.22 |  | <0.0001**^+^** |
|  |  |  |  |  |  |  |  |
| *ELC* Early Learning Composite, *EL* Expressive Language*, RL* Receptive Language  ^a^Standardized beta coefficients from linear regression models. Reference groups for Sibling sex and Group are female (vs. male) and HR-ASD (vs. HR-NoASD), respectively.  **^+^**significant at p ≤ 0.008 after Bonferroni correction for main and interacting  effects of proband score (6 comparisons). | | | | | | | |

**Figure S2. Scatterplots: MSEL Age-Adjusted Scores**

Raw scatterplots between proband VABS-II EL, RL and Sibling MSEL EL, RL age-adjusted scores. Pearson’s correlations are shown for the full group (gray), concordant pairs (red), and discordant pairs (blue). Significance is indicated as follows: * ≤0.05, ** ≤0.01, ***≤0.001
